# Improved Analysis of Glyphosate, Aminomethylphosphonic Acid, and Other Highly Polar Pesticides and Metabolites via the QuPPe Method by Employing Ethylenediaminetetraacetic Acid and IC-MS/MS

**DOI:** 10.1021/acs.jafc.4c08461

**Published:** 2025-01-15

**Authors:** Ann-Kathrin Schäfer, Walter Vetter, Michelangelo Anastassiades

**Affiliations:** 1 EU-Reference Laboratory for Pesticides Requiring Single Residue Methods (EURL-SRM), Chemisches und Veterinäruntersuchungsamt Stuttgart, Fellbach D-70736, Germany; 2 Institute of Food Chemistry (170b), University of Hohenheim, Stuttgart D-70599, Germany

**Keywords:** anionic polar pesticide, food, IC-MS/MS, glyphosate residues, EDTA, QuPPe

## Abstract

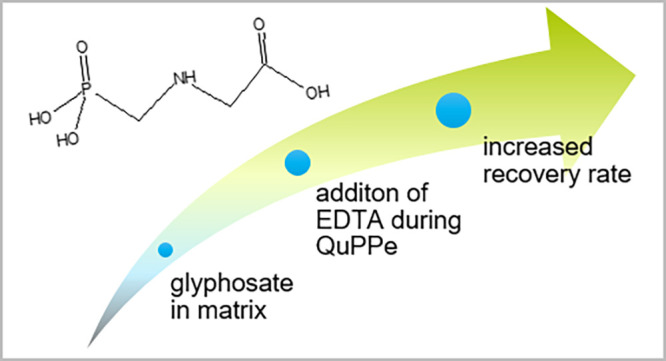

The quantification of glyphosate (Gly) and its metabolite aminomethylphosphonic acid (AMPA) in food is often impaired by matrix components. Specifically, interaction between the analytes and natural matrix components in food leads to reduced analyte recovery rates. Here, we studied how the addition of ethylenediaminetetraacetic acid (EDTA) impacted the QuPPe recovery rates of Gly and its metabolite in eight mostly problematic matrices using tandem mass spectrometry. QuPPe recovery rates of glyphosate and AMPA could be increased dramatically, e.g., in milk from 29 to 93% and 18 to 93%, respectively. Additionally, isotopically labeled internal standards (IL-ISs) effectively compensated for the losses, resulting in IL-IS-corrected recoveries of ∼100% in all cases. The recovery-suppressing effect of metal cations was confirmed in metal-addition experiments, where Gly was more affected through the addition of calcium ions and AMPA through the addition of iron ions to the sample. Overall, the use of EDTA helped to overcome the most serious challenges in the analysis of Gly and AMPA via the QuPPe method without compromising ion chromatography or mass spectrometric detection of these two as well as of 11 additional anionic analytes also covered by this methods.

## Introduction

**Figure 1:**
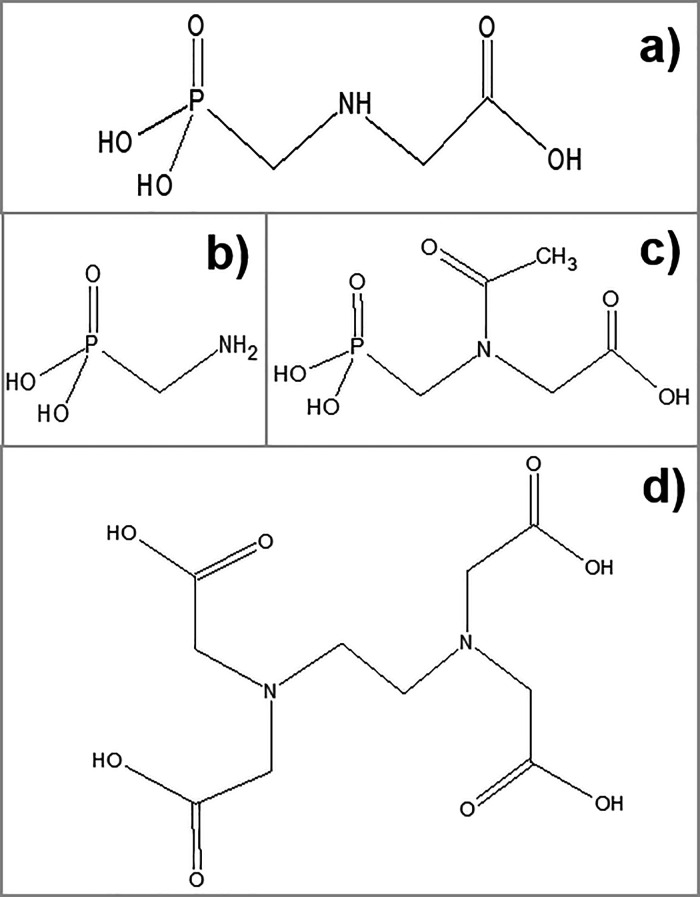
Chemical structures of (a) the herbicide glyphosate (Gly), (b) its metabolite aminomethylphosphonic acid (AMPA), and (c) its metabolite *N*-acetyl-glyphosate (NAGly) as well as that of (d) the common chelating agent ethylenediaminetetraacetic acid (EDTA) that was used for complexation.

Glyphosate (Gly) (Figure 1a) is a nonselective phosphonate herbicide, which affects a broad spectrum of weeds by targeting the shikimic acid pathway and thus suppresses the ability of plants to synthesize amino acids.^1,2^ In agriculture, Gly is primarily used for controlling weeds before the seeding of crop plants, as well as for the postemergence control of weeds growing around perennials.^1,3^ With Gly being the most widely used herbicide,^2,4^ analysis of its residues in food and environmental samples is essential. Direct analysis of Gly and related polar pesticides can be accomplished via liquid chromatography or ion chromatography in combination with tandem mass spectrometry (LC-MS/MS or IC-MS/MS).^5,6^ Prior to instrumental analysis, extraction can be carried out using the quick polar pesticides (QuPPe) method, developed by the EU Reference Laboratory for pesticides requiring single residue methods (EURL-SRM).^5^

Gly bears a secondary amino group with basic properties (p*K*_a_ 11.0), a carboxylic acid moiety of intermediate acidic strength (p*K*_a_ 2.29), and a diprotic phosphonic acid moiety with two dissociable protons (p*K*_a_ < 1 and 5.96).^7^ The two main metabolites of Gly perspectively relevant for enforcement within the European Union (EU), aminomethylphosphonic acid (AMPA, Figure 1b) and *N*-acetyl-glyphosate (NAGly, Figure 1c), maintain the phosphonic acid moiety, and NAGly also maintains the carboxylic acid moiety. In either case, the anionic microspecies can be chelated by metal cations (Table 1 and Table S1, Supporting Information), which may affect the recovery of the analytes.^7−12^ Specifically, the most stable complexes of Gly with common metals are those with Fe^3+^ and the least stable are those with Ca^2+^ and Mg^2+^ (Table 1).^9−12^ At the same time, many food commodities contain high calcium levels (e.g., ∼1200 mg/kg calcium in cow’s milk,^13-15^Table S2, Supporting Information). Although the extent to which Gly is bound to metals in plant phloem was reported to be limited, it is conceivable that metal ions that are only partly chelated by matrix components, and thus still possess free valences, could possibly interact with Gly and its metabolites through mixed-ligand complexes.^14−16^ This could thereby decrease the recovery rates of the target analytes in their free form.^16^ Such recovery losses have been reported for direct determination methods primarily employing aqueous extraction solvent and/or methanol.^14,15,17^ However, alternative studies using, e.g., derivatization, immune assays, or direct determination after ultrafiltration of milk, did not report such losses in food.^18−20^ Irrespectively, the presence of metal ions can reportedly also inhibit the derivatization of Gly and AMPA in water and aqueous solutions.^21,22^ Therefore, interactions of Gly and other chelating analytes with matrix components, including the associated recovery losses, should be taken into account in the analysis.

**Table 1:** Logarithmic Stability Constants of Complexes Formed between Glyphosate or AMPA with Metal Ions in Comparison to Those of EDTA, Citric Acid, and Oxalic Acid^a^

metal	glyphosate ^1^	AMPA ^1^	EDTA^*2*^	citric acid^2^	oxalic acid^3^
Ca^2+^	3.3	1.6	12.4	4.9	2.67^11^ to 3.44^12^^,^^b^
Mg^2+^	3.3c	1.9	10.6	4.9	3.0 (2.39^11^)
Fe^2+^	6.9	c	16	6.1	>4.7
Fe^3+^	16.1^10^	c	27.7	13.2	9.4
Cu^2+^	11.9	8.1	20.5	10.9	6.3
Zn^2+^	8.4	4.9	18.2	6.1	4.9
Mn^2+^	5.5	3.6	15.6	5.0	3.9

a Data shown was adopted from a compilation by Mertens et al.,^9^ except where otherwise stated. Other values were collected from Popov et al.,^10^ Urbańska,^11^ and Berland et al.^12^ The shown stability constants were reportedly measured by potentiometric pH titration at ionic strength of (superscript 1) I = 0.1 M KNO_3_; (superscript 2) I = 0; and (superscript 3) no information given.

b According to^11^ the stability constant of Ca^2+^-oxalate is greater than that of Mg^2+^-oxalate.

c No information available; stability constants expectedly lower than those of glyphosate.

Recovery losses occurring when employing the QuPPe or methanol/water-based extraction with direct determination can be overcome by spiking the sample with a competitor, i.e., a compound having an even stronger chelating capacity. Based on the metal complex stability constants of Gly and AMPA (Table 1), this is achieved with the common chelating agent EDTA (Figure 1d), which has already been used as a chelating competitor.^14,15^ The complex stabilities of EDTA also typically exceed those of the natural matrix components citric and oxalic acids (Table 1).^9−12^ Accordingly, the addition of EDTA has already become normative for the derivatization of Gly in water according to DIN ISO 16308.^23^ EDTA has been further employed for improved extraction and analysis of nonderivatized Gly, glufosinate, and AMPA in soybean, corn, and milk.^14,15^ Preliminary results indicated that implementing EDTA into the QuPPe method can improve the recovery rates of Gly and its metabolites in some matrices. Matrix-induced signal suppressions can further drop the overall sensitivity and lead to conditions under which the identification criteria cannot be fulfilled.^24^

The goal of the present study was to thoroughly examine the benefit of adding EDTA on the QuPPe recovery rates of Gly, AMPA, and NAGly in various problematic food matrices with the determination being conducted via ion chromatography tandem mass spectrometry (IC-MS/MS) according to Schäfer et al.^25^ By utilizing the QuPPe method as opposed to single residue or group-specific residue methods, the determination of Gly, AMPA, and NAGly can be included within a multimethod covering more than 50 analytes, including 14 anionic compounds analyzed by IC-MS/MS.^5,24^ Two procedures were studied to overcome the low recovery rates of Gly and its metabolites. Furthermore, the impact of the metal ion content and the addition of EDTA were investigated in a model experiment. All experimental conditions were chosen to reflect the real scenarios found in food samples. Also tested was the effect of EDTA on the recovery rates of other polar analytes as well as the effect of adding calcium and iron ions to the samples before extraction. Additionally, ion conductivity chromatograms were recorded to show the presence of free and complexed EDTA.

## Materials and Methods

### Chemicals and Consumables

Methanol and acetonitrile (ACN), of HPLC quality, were purchased from Supelco Merck (Darmstadt, Germany). LC-MS-grade ACN was ordered from Chemsolute Th. Geyer (Renningen, Germany)/Supelco Merck (Darmstadt, Germany)/Biosolve Chemicals (Valkenswaard, The Netherlands) (different sources due to supply difficulties). Formic acid (HPLC grade) was provided by Merck (Darmstadt, Germany). The C_18_ sorbent (POLYGOPREP 300-30 C18, particle size 30 μm and pore size 300 Å) originated from Macherey-Nagel (Düren, Germany), while CaCl_2_ and FeCl_3_ (p.a.) were purchased from Sigma-Aldrich (Darmstadt, Germany). Ethylenediaminetetraacetic acid tetrasodium tetrahydrate (Na_4_EDTA·4H_2_O, p.a. grade) was delivered by Millipore Merck (Darmstadt, Germany). An EDTA solution (10%, w/w) was prepared by placing 15.85 g of Na_4_EDTA·4H_2_O and approximately 80 mL of distilled water into a 100 mL volumetric flask with a stopper. After thorough shaking, the flask was filled up to 100 mL with distilled water. A sample material for the experiments was purchased from the local market. Standards of native pesticides (Gly and its metabolites) were ordered from Dr. Ehrenstorfer (Augsburg, Germany) and HPC (Cunnersdorf, Germany). Standards of the analytes further included within the QuPPe method were purchased from Dr. Ehrenstorfer (Augsburg, Germany), HPC (Cunnersdorf, Germany), Toronto Research Chemicals (TRC)/LGC (Teddington, England), Merck/Sigma-Aldrich (Darmstadt, Germany), Merck (Darmstadt, Germany), ASCA (Adlershof, Germany), and LGC/Fluka (Teddington, England). Isotopically labeled internal standards (IL-IS) originated from Toronto Research Chemicals (TRC)/LGC (Teddington, England). Stock solutions were prepared in water containing 10% (v/v) ACN at 1000 μg/mL. Working standards of the three components in a mixture, at, e.g., 10 μg/mL each, were prepared by diluting the stock solutions with 10% ACN in water.

Fifty-milliliter centrifuge plastic (polypropylene) tubes with screw caps were purchased from Sarstedt (Nümbrecht, Germany), and 10 mL centrifuge plastic (polypropylene) tubes with screw caps were obtained from Simport Scientific (Saint-Mathieu de Beloiel, Quebec, Canada).^5,21^ Five-milliliter syringes (polypropylene) and 0.2 μm-pore size syringe filters with hydrophilized polytetrafluoroethylene (H-PTFE) were ordered from Macherey-Nagel (Düren, Germany). Plastic (polypropylene) autosampler vials (2 mL) originated from Klaus Ziemer (Langerwehe, Germany).^5,26^ It is important to note that glass vials should not be used because of interactions with Gly and other analytes.^5^

### Instrumentation and Analytical Method: IC-MS/MS

A Dionex Integrion HPIC (with autosampler Thermo Fisher Scientific Dionex AS-AP; Thermo Fisher Scientific, Waltham, MA, USA) was interfaced to an AB Sciex QTrap 5500 mass spectrometer (AB Sciex, Framingham, MA, USA). The IC was operated with Chromeleon software (Thermo Fisher Scientific, Waltham, MA, USA), and the MS was operated with Analyst Software (AB Sciex, Framingham, MA, USA). ACN was introduced to the eluent via a T-piece just before the ion source using an AXP-MS auxiliary pump (Thermo Fisher Scientific, Waltham, MA, USA).^25^ Additionally, an external Reglo Digital pump (Ismatec/Cole-Parmer, Vernon Hills, IL, USA) was used for regeneration of the suppressor. Separations were performed with an AS19 2x 250 mm column in combination with an AG19 2x 50 mm precolumn (Thermo Fisher Scientific, Waltham, MA, USA). After 8 min at 15 mM,^5,6,25^ the KOH concentration was increased to 36 mM over 5 min and again held for 8 min; it was then increased to 70 mM over 0.5 min and held for 3.5 min.^5,6,25^ At this point (25.5 min), the KOH concentration was reduced to 15 mM and maintained at this level for 4.5 min for re-equilibration.^5,6,25^ The injection volume was set at 5 μL, the flow rate at 0.3 mL/min, and the oven temperature at 32 °C.^5,6,25^ More information is given in Table S3 (Supporting Information).

### Sample Preparation: Extraction Procedure

Samples were prepared following the procedures of the QuPPe method for matrices of plant origin (QuPPe-PO) and for matrices of animal origin (QuPPe-AO), with modifications described in each experiment.^5,26^ In brief, 2–10 g of sample homogenate was spiked with isotopically labeled internal standards (IL-IS) and, depending on the water content, supplemented with an appropriate amount of deionized water to reach a total water content of close to 10 mL (Table S4). The solution was then extracted with 10 mL of acidified methanol (containing 1% formic acid), followed by mechanical shaking, centrifugation, and filtration with 0.2 μm-pore size syringe filters.^5,26^ For matrices rich in proteins and/or lipids (here all matrices, except cucumber and carrot), proteins and lipids were removed from the QuPPe sample extract as described below as well as in Table S4.^5,26^ Depending on the water content of the matrix, the total volume was adjusted to 20 mL by adding distilled water before the extraction.

Blank matrix extracts for calibration solutions were prepared without the addition of IL-IS, using matrices without detectable levels of any of the analytes of interest. Further modifications are described below.

### Recovery Experiments with and without Addition of EDTA

The impact of adding EDTA during extraction on recovery rates was examined in matrices being exceptionally rich in iron and/or calcium and/or other divalent metal ions, i.e., milk (full-fat), liver (homogenized), sesame (ground into powder), wheat (milled into flour), infant food formula (dry, based on cow’s milk), cocoa powder (milled partly defatted nibs), and lentils (milled into flour). Additionally, cucumber was chosen as a matrix low in metal ions.^13^ With this selection, six (out of eight) commodity groups of the SANTE quality control procedures^19^ were represented (Table S4).

#### General Procedure

The samples (10 g milk, liver, or cucumber; 5 g sesame, wheat, cocoa bean, or lentils; 2 g infant food formula) were weighed into 50 mL centrifugal plastic tubes and spiked in triplicate with 1 μg of Gly, AMPA, and NAGly at 0.1 mg/kg (milk, liver, or cucumber), 0.2 mg/kg (sesame, wheat, cocoa, or lentils), and 0.5 mg/kg (infant food formula) and the corresponding isotopically labeled internal standards (IL-IS) (Table S4).^5^ Further possible metabolites of Gly were not included in this study, as they are not relevant for enforcement in the EU. According to the QuPPe method,^5,26^ 1 to 10 mL of water (Table S4) was added to adjust the total water content to ca. 10 g (based on the typical natural water contents listed in Souci et al.^13^ as rounded in the QuPPe method^5,26^). Subsequently, 10 mL of methanol containing 1% formic acid and 1 mL of a 10% aqueous EDTA solution were added. This corresponds to 100 mg or 0.34 mmol of EDTA (see also Table S5 in the Supporting Information). Where no EDTA was added, 1 mL water was added instead.

Due to the high metal cation content in sesame, two further variants were tested: (a) reducing the sample weight from 5 to 2 g (variant 1 for sesame) and (b) using double the amount of EDTA (variant 2 for sesame) (Table S5).

During extraction (general procedure, and variants 1 and 2 for sesame), all samples were supplemented with 100 μL of formic acid to lower the pH values consistent with the isoelectric point of many proteins (and thus promote precipitation) and to omit the degradation of certain pesticides (in particular ethephon).^5^ The addition of extra 100 μL of formic acid was skipped, where no EDTA was added. After freeze-out and centrifugation, further proteins were precipitated by diluting the supernatant with ACN 1:1 (except for cucumber where no dilution is foreseen).^5,26^ This step was combined with dispersive solid phase extraction (dSPE). Specifically, 50 mg of octadecylsilane (ODS) sorbent and 2 mL of ACN were placed in a 10 mL plastic centrifuge tube followed by the addition of 2 mL of the supernatant raw extract.^5,26^ This step helped to remove the bulk of the coextracted fat/oil in the raw extract (except for cucumber).^5,26^ After centrifugation, the supernatant was filtered using a syringe and 0.2 μm pore size syringe filters.^5,26^ The final extracts of the experiments were 5-fold diluted by placing a 200 μL aliquot of the extracts in a 2 mL plastic auto sampler vial and adding 800 μL of ultrapure water, solvent, and/or standard mixture (total volume of 1000 μL). After thorough shaking, the analytes were quantified by IC-MS/MS against a four-point calibration in blank extracts of the respective matrices at 30, 60, 100, and 120% of the spiked recovery level. Separate matrix-matched blank extracts (and calibration curves) were prepared for each matrix and each extraction variant (e.g., with or without the addition of EDTA). Furthermore, the nonspiked extract was measured to consider any background signals and the linearity was proven to be satisfactory in the investigated range.

### Recovery Experiments with Model Matrices

Recovery rates were determined in the model matrix (carrot) and supplemented with calcium or iron ions directly spiked into the sample. In triplicate, five levels of solid CaCl_2_ (14, 28, 42, 56, or 69 mg of CaCl_2_ corresponding to 5, 10, 15, 20, and 25 mg of Ca^2+^) were added to 10 g of carrot homogenate in a 50 mL plastic centrifuge tube (Table S6, Supporting Information), with, e.g., 10 mg of Ca^2+^ roughly corresponding to the natural content of Ca^2+^ in 10 g of milk (Table S2). In a similar manner, FeCl_3_ solution was added to reach a level of either 0.5 mg (50 μL of a 10 mg/mL solution) or 1 mg (100 μL of a 10 mg/mL solution) of Fe(III) in 10 g of carrot homogenate (*n* = 3) in a 50 mL plastic centrifuge tube (Table S6). Next, the samples were spiked with Gly, AMPA, and NAGly (each 1 μg) at 0.1 mg/kg (without IL-IS). After adding the extraction solvent (methanol containing 1% formic acid), the sample solution was supplemented with 1 mL of aqueous EDTA solution (10%) and 100 μL of formic acid. In samples where EDTA solution was substituted with 1 mL of water, formic acid (100 μL) was also omitted. As the carrot sample does not contain substantial amounts of protein or lipids, the cleanup step combining dSPE with ACN dilution for protein precipitation was skipped.^13^ The final extracts were measured following a 5-fold dilution (800 μL of ultrapure water, solvent, and/or standard mixture added to 200 μL of extract in 2 mL plastic autosampler vials, see above). Quantification was performed against a two-point calibration of the analytes in blank carrot extract with concentrations at 60 and 120% of the spiked recovery level. Separate matrix-matched blank extracts were prepared for each variant (i.e., with or without adding Ca^2+^ or Fe^3+^ at the respective concentration and with or without EDTA). Furthermore, the nonspiked extract was measured to consider any background signals and the linearity was proven to be satisfactory in the investigated range.

### Studying the Impact of EDTA in Ion Chromatography (IC)

The effect of EDTA on IC separation was monitored by injecting QuPPe extracts of pure water, milk, and liver extracted with and without EDTA (see above); carrot extract was spiked with 10 mg of Ca^2+^ per 10 g of analytical portion and extracted with and without EDTA (see above). A conductivity detector was used for these measurements. In addition, a standard solution of 200 μg/mL EDTA in water (i.e., at a much lower amount than in the principal procedure, see below) was tested in this experiment.

### Note on Nomenclature and Use of Terms regarding Recovery Rates, according to SANTE^24^

Nomenclature within this study is used in accordance with the guidelines, where the “absolute recovery” is defined as the “the proportion of analyte (yield) remaining at the end point of the final determination following its addition (usually to a blank analytical test portion) prior to extraction”.^24^ The “absolute recovery” is distinguished from the “(apparent) recovery”, which takes into account the whole analytical method including any mathematical correction for method bias (e.g., via recovery factors, other suitable calibration approaches or using IL-IS).^24^ IL-IS-corrected recovery figures are also considered apparent recoveries.^24^ “Apparent recovery” is mostly referred to as “recovery” in this guidance document.^24^

## Results and Discussion

### Measures for Improving the QuPPe Recovery Rates of Glyphosate (Gly) and Its Metabolites (AMPA and NAGly)

The addition of EDTA according to the principal procedure (2–10 g of sample spiked with 1 μg of Gly, AMPA, and NAGly and the corresponding IL-IS in ∼20 mL solution, 1 mL of 10% EDTA solution, see the experimental part) improved the absolute recovery rate (as defined in the SANTE quality control procedures document^24^) in cucumber, milk, liver, sesame, lentils, cocoa bean, wheat, and infant food.

### Absolute Recovery Rates Employing the QuPPe Method (without Corrections by IL-ISs)

#### Glyphosate (Gly)

**Figure 2:**
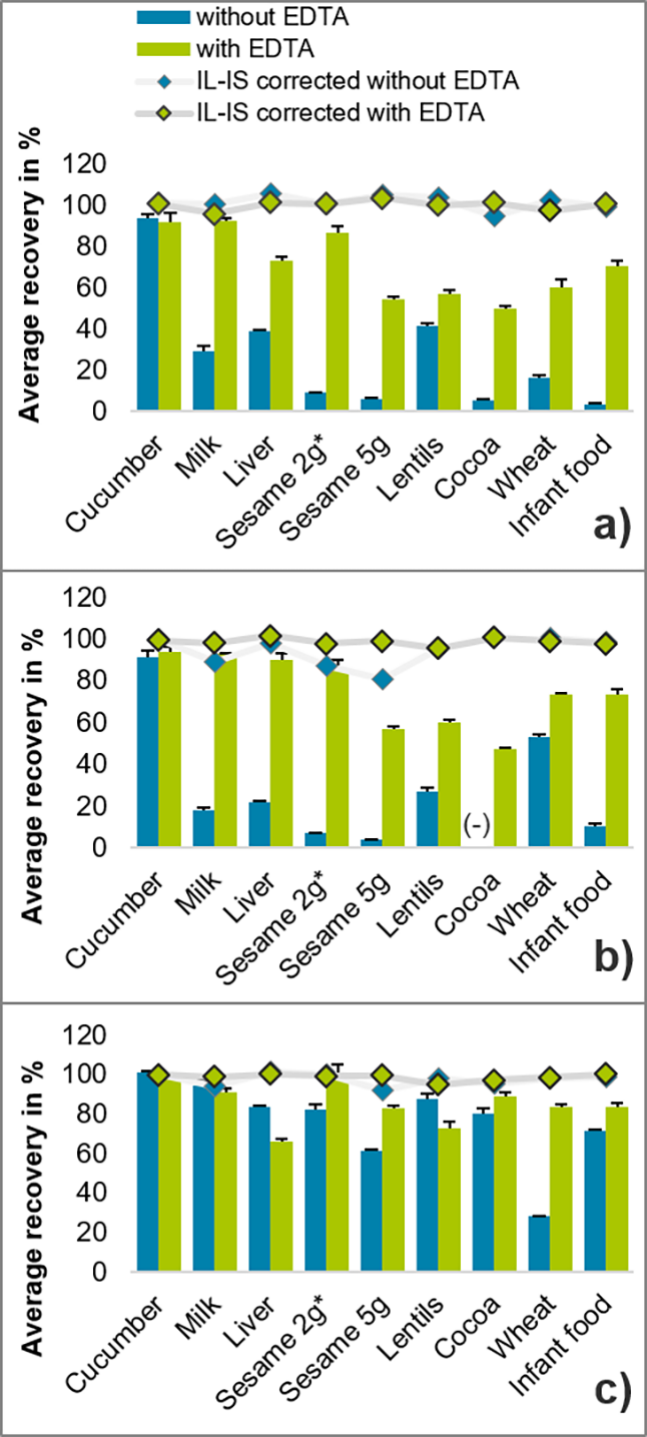
Recovery rates (*n* = 3) (mean values and standard deviations of three analyses based on “experiment 1” (recovery experiments with and without EDTA addition)) of (a) glyphosate (Gly), (b) aminomethylphosphonic acid (AMPA), and (c) *N*-acetyl-glyphosate (NAGly) without and with EDTA (bars: no correction by isotopically labeled internal standards (IL-ISs); diamonds: after IL-IS correction). Data is given in Table S7 in the Supporting Information. *Variant 2 for sesame.

With the exception of cucumber (which is known to be low in metal ions, proteins, and other constituents that could potentially lead to recovery losses), the average absolute recovery rate of Gly without EDTA in the eight tested matrices was generally <50 and in some cases even <10% (Figure 2a, blue bars). The addition of EDTA was followed by a strong increase in the absolute recovery rate in all matrices to generally >50% (Figure 2a, green bars). In fact, the addition of 1 mL of 10% EDTA solution ensured absolute recoveries of Gly exceeding the threshold of 30% stipulated by the SANTE quality control procedures document.^24^

#### Aminomethylphosphonic Acid (AMPA)

The main metabolite Gly showed a similar tendency (Figure 2b). Without EDTA and excluding cucumbers, the average of the measured absolute recovery rate of AMPA ranged from not determinable (due to the absence of a signal in the extracts of spiked cocoa bean samples) to 53% in wheat (Figure 2b, blue bars). In cocoa beans, even the IL-IS of AMPA was not detectable. Using EDTA, the average absolute recovery rate of AMPA generally exceeded 47%. Remarkably, EDTA increased the absolute recovery rate of AMPA in milk from 18 to 93% (Figure 2b, green bars). In cocoa beans, the average absolute recovery rate was determined at 47% when EDTA was added.

#### *N*-Acetyl-glyphosate (NAGly)

Except for wheat (28%), the absolute recovery rates of NAGly (Figure 2c) were already high without EDTA (Figure 2c). However, the addition of EDTA further improved the absolute recovery rate to values >70% (Figure 2c). Only in the case of liver and lentils were the absolute recovery rates ∼10% lower than those without EDTA, but still well within the acceptable range (Figure 2c).

#### Overall Assessment and Consequences

The relative standard deviations of the triplicate analyses were <5%, except for Gly in sesame without EDTA and for Gly and NAGly in cocoa without EDTA (Figure 2), which indicated a good reproducibility of the procedure. Despite the addition of EDTA, the absolute recovery rates of Gly and AMPA remained comparably low (47–60%) in sesame, lentils, and cocoa. According to the literature, sesame contains the highest amounts of calcium of all tested matrices (Table S2).^13^ Therefore, the sample weight of sesame was lowered from 5 to 2 g (see Materials and Methods, variant 1 for sesame), which in turn increased the absolute recovery rate of both pesticides to >80% (Figure 2a,b). In contrast, doubling the amount of EDTA and maintaining the higher sample amount (5 g) did not improve the absolute recovery rate of Gly and AMPA (see Materials and Methods, variant 2 for sesame) (Figure S1). This can be attributed to an oversaturation of EDTA (precipitation was noticed). It is further known that the presence of oxalate lowers the amount of bioavailable and free calcium and magnesium in sesame and cocoa (Table S2).^13,27−29^ This extra complexation of alkaline earth metals by matrix components reduces the required amount of EDTA.

### IL-IS-Corrected Recovery Rates Using the QuPPe Method

With the exception of cocoa beans, where no recovery rates could be determined since neither analyte nor IL-IS was detected (see above), IL-IS-corrected recovery rates ranged from 80 to 110%, even without EDTA. IL-IS-corrected recovery rates of Gly and AMPA were comparable irrespective of the addition of EDTA (Figure 2, diamonds above the bars). However, for cocoa, the use of EDTA proved essential for the analysis of Gly and AMPA as it enabled the detection of measurable signals. This is also potentially valid for other commodities not covered in this study. It is well assumable that for matrices where absolute recovery rates for AMPA and Gly without addition of EDTA were ≤30% (such as milk, liver, sesame, lentils, cocoa, and infant food; Figure 2a,b), the implementation of EDTA addition in the QuPPe method will reduce the risk of false negative results (especially at residue levels of <0.1 mg/kg, which was the concentration spiked in the present experiment).

Admittedly, it is challenging to estimate the extent of the matrix influence on recovery rates to avoid false negative assessments. However, the use of IL-ISs during extraction is beneficial not only for correcting for bias including poor recoveries but also for recognizing strong signal suppressions and/or poor recovery rates. Given these promising results from the addition of EDTA during extraction, this approach was expanded to further highly polar analytes.

### Effect of EDTA on Recovery Rates of Other Polar Analytes Using the QuPPe Method

Contrary to Gly and AMPA, only moderate (ethephon) to negligible (fosetyl, 2-hydroxyethylphosphonic acid (HEPA), glufosinate, 3-methyl-phosphinicopropionic acid (MPPA), and *N*-acetyl-glufosinate) improvements in the absolute recovery rates (without correction by IL-ISs) were observed. However, the absolute recovery rates for these analytes were already >70% without EDTA (detailed data is given Table S8, Supporting Information). Accordingly, an effect in the same dimension as that for Gly and AMPA cannot be expected. Further experiments with various matrices (not shown here) showed that the performance criteria for pesticide residue analysis laid down in DG-SANTE were fulfilled both with and without IL-IS correction.^24,25^ Accordingly, Gly and AMPA remained the two most critical compounds. By employing the QuPPe method, Gly and its metabolites can be included in a multiresidue method, which is expected to become normative in the EU during 2025, following interlaboratory validation studies on several matrices of plant and animal origin.

### Model Experiments Involving Spiking of Calcium or Iron Ions at Various Concentrations onto the Carrot Matrix

The effect of adding Ca^2+^ (added as CaCl_2_ salt) or Fe(III) (added as FeCl_3_) on the recoveries of Gly and AMPA was further tested in a subsequent experiment, with the QuPPe extractions being performed both with and without addition of EDTA. Carrot homogenate was selected for this experiment because of the known low natural level of both metal cations.^13^ In the first step, the recovery rates were determined without corrections by means of IL-ISs (see Materials and Methods).

#### Glyphosate (Gly) and Aminomethylphosphonic Acid (AMPA)

**Figure 3:**
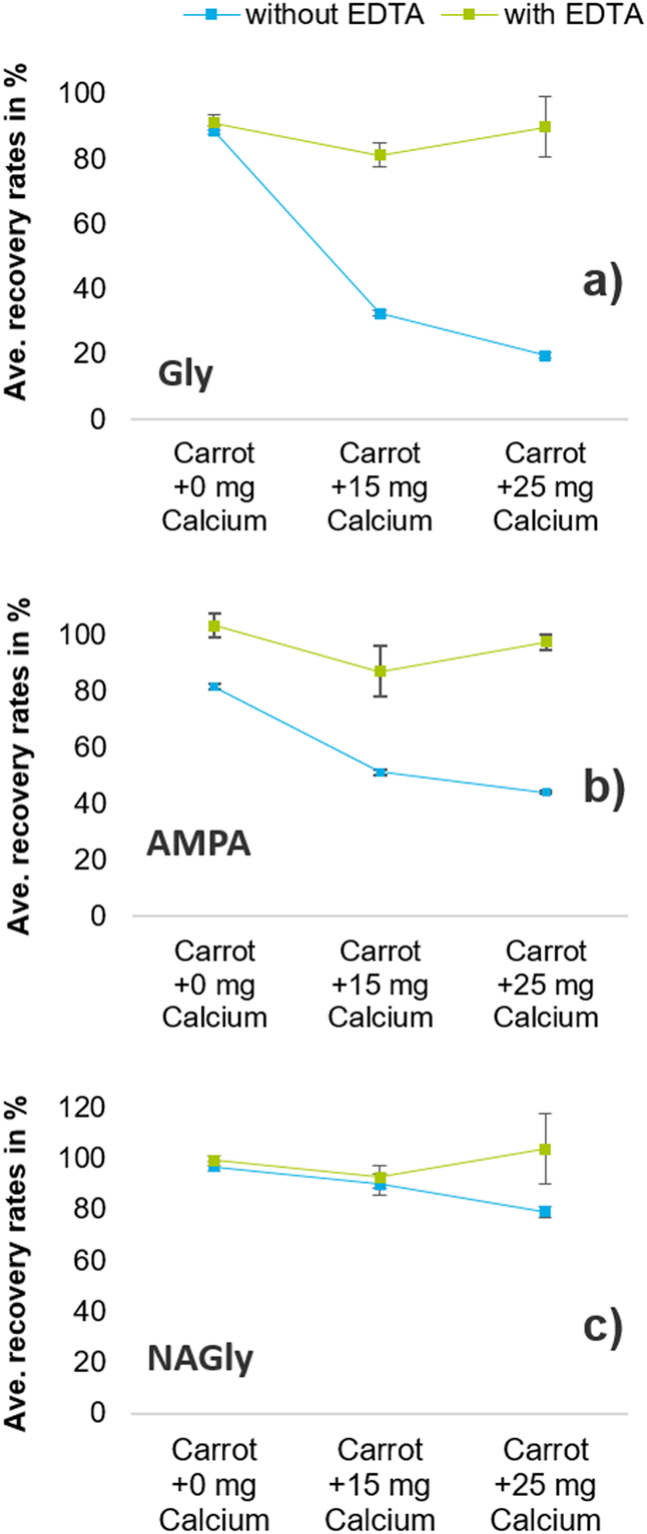
Average absolute recovery rates (without IL-IS correction) of glyphosate (a), AMPA (b), and NAGly (c) in carrot after adding different amounts of Ca^2+^, with (green squares) and without (blue squares) addition of EDTA during extraction.

**Figure 4:**
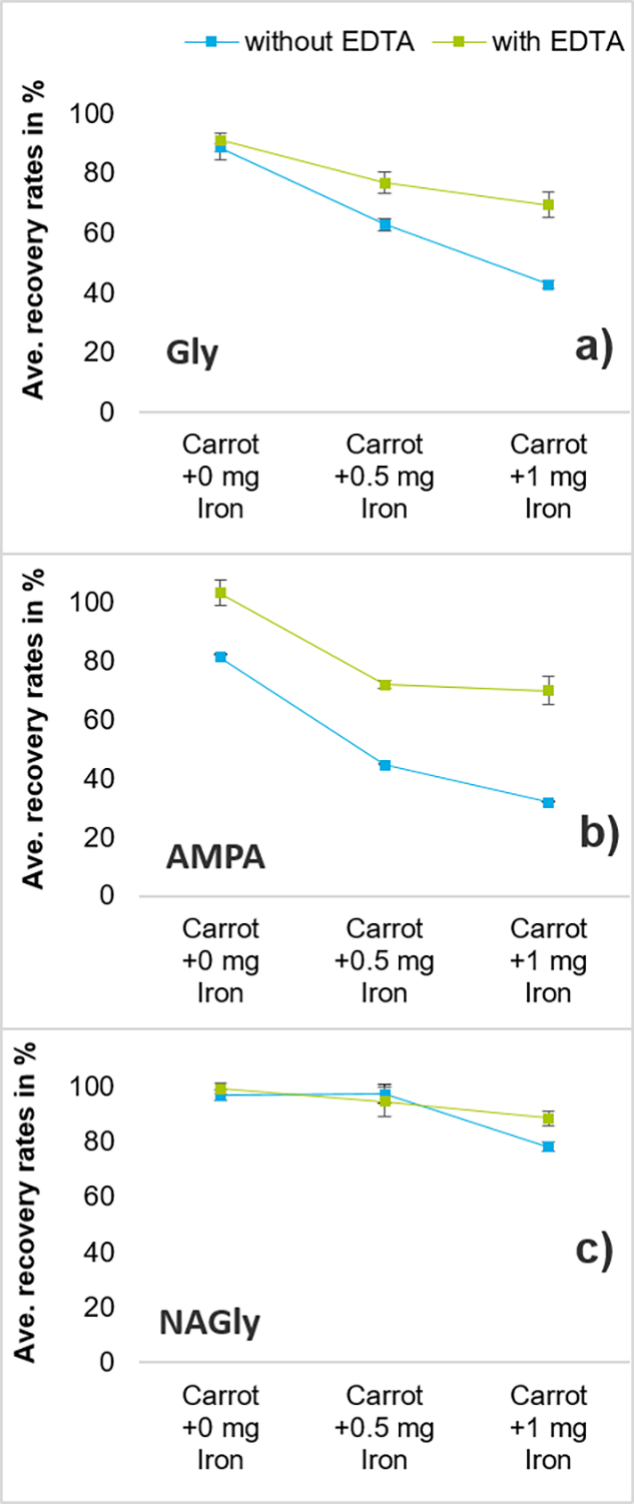
Average absolute recovery rates (without IL-IS correction) of glyphosate (a), AMPA (b), and NAGly (c) in carrot after adding different amounts of Fe^3+^, with (green squares) and without (blue squares) addition of EDTA during extraction.

Fe(III) was preferred over Fe(II) for the experiment, as it usually forms more stable complexes (Table 1). Without added metal ions, the absolute recovery rate of Gly was similarly high with (91%) and without EDTA (89%) (Figures 3a and 4a). However, the absolute recovery rate dropped below 70% when Ca^2+^ (≥10 mg) or Fe^3+^ (≥0.5 mg) was added. Still, the absolute recoveries improved again by adding EDTA (Figures 3a and 4a).

Without added metal ions and EDTA, the absolute recovery rate of AMPA was relatively high (83%) and could be further increased to 98% with EDTA (Figures 3b and 4b). Addition of Ca^2+^ affected the absolute recovery rates of Gly (Figure 3a) more than those of AMPA (Figure 3b), which was in line with reported complex stability constants (Table 1) and confirmed that Ca^2+^ complexes more strongly with Gly than with AMPA.

By contrast, AMPA was more affected by the addition of Fe^3+^ than by Gly (Figure 4b). Despite the very high stability constant of the Fe^3+^/EDTA complex (Table 1), EDTA could not increase the absolute recovery rates of Gly and AMPA to above 70% when the sample portions were spiked with the higher tested dose of 1 mg of Fe^3+^ (Figure 4a,b).

#### *N*-Acetyl-glyphosate (NAGly)

As expected, NAGly absolute recovery rates only moderately dropped upon addition of Fe^3+^ or Ca^2+^ to the carrot homogenate (Figures 3c and 4c). Also, the benefit of adding EDTA was rather small. Both observations confirmed the assumption that polar pesticides with weak tendencies to form metal chelates were less affected by the presence of metal ions. However, in two of the tested commodities (i.e., sesame and wheat) absolute recovery rates of <70% were observed. In both cases, the addition of EDTA markedly increased the absolute recovery rates of NAGly.

#### Overall Assessment and Consequences

In unspiked carrot, which is known to be low in metals, the absolute recovery rates of Gly, AMPA, and NAGly were high (89, 81, and 97%) without using EDTA. Similar observations were made with cucumber and other fruits and vegetables not shown here. In such matrices with a low metal content, the use of EDTA is, therefore, not essential. In general, it is difficult to set an (absolute) recovery threshold below which EDTA should be used, especially since the use of IL-ISs typically corrects for the bias.

Unfortunately, spiking experiments with model matrices cannot fully simulate the situation in real samples because they usually contain strongly varying ratios of diverse matrix components that could potentially influence both metal and analyte complexation. Possible candidates include other metals, organic acids, amino acids, catechins, and especially proteins, which are only partly soluble in QuPPe extracts.^15^ Furthermore, according to Harris et al., other biological agents in plant matrices, such as nicotianamine, are much stronger chelators than Gly, leading to mostly unbound Gly in plant phloem.^16^ For instance, the model experiment with carrot and 10 mg of Ca^2+^ resulted in absolute recovery rates (62% AMPA and 51% Gly) higher than those obtained in samples naturally rich in metal ions and protein (Figure 2a,b). Obviously, other metals, proteins, and/or other components with the potential to form complexes with analytes may also affect absolute recoveries in matrices rich in, e.g., calcium and/or iron (Table S2). The binding rate of Gly and other (natural) chelating agents with metal ions in plants was shown to be largely driven by competition.^16^ Additionally, mixed-ligand binding of Gly with proteins and the associated precipitation of those components will expectedly lead to recovery losses. In preliminary experiments performed on low protein matrices (e.g., cucumber), even higher losses were partly observed when QuPPe extracts generated without using EDTA (as foreseen by the method) were diluted with ACN (not foreseen) in both the presence or absence of the C18 sorbent. Further investigations are required to elucidate the competitive binding environment during extraction and cleanup, specifically for protein- and lipid-rich samples. In this context, spiking of proteins would be a suitable experiment for further investigating the mechanisms leading to recovery losses. Irrespectively, results reported in this study strongly suggest that EDTA effectively displaces Gly from interaction sites, thus improving recovery rates.

### Observations of EDTA in IC

**Figure 5:**
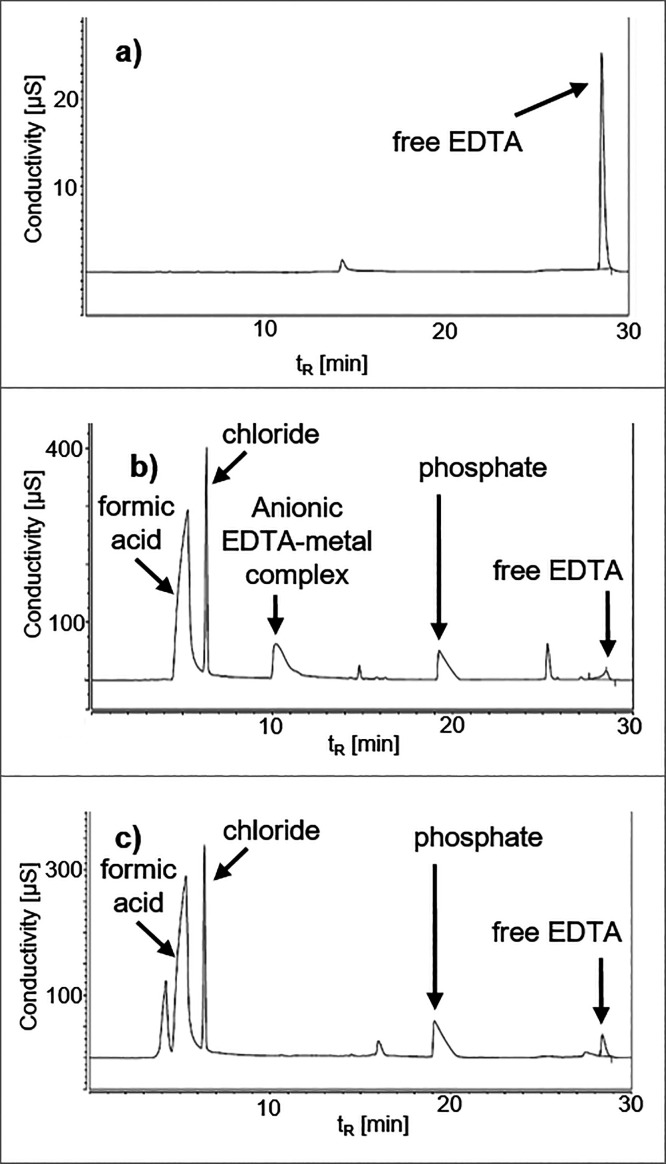
Conductivity chromatograms of a calibration standard with 200 μg/mL EDTA in water (a) and undiluted sample extracts of milk (b) and liver (c) showing free EDTA at a retention time (*t*_R_) of about 28.5 min and the EDTA-complexed metal at a *t*_R_ of 10.5 min, as well as further identified peaks.

Conductivity chromatograms were recorded for three matrix extracts (milk, liver, and carrot; see Materials and Methods), with water serving as the reference standard. In either case, a signal was detected at the retention time (*t*_R_) of EDTA of ∼28 min (Figure 5). This indicated that a share of the excessively added EDTA was present as free anions in the final QuPPe extracts (Figure 5). In the case of liver, a potential anionic EDTA–metal complex was detected at *t*_R_ ∼ 10 min (Figure 5b).

Similarly, the extracts of the model matrix, carrot, featured an anionic EDTA complex. The addition of both CaCl_2_ and EDTA to the carrot extract also resulted in a peak at ∼10 min, as detected in liver (see above). Likewise, this signal was attributed to a soluble anionic [Ca^2+^-EDTA]^2–^ complex (Figure S2b). However, in this case, free EDTA was not detected after 28 min. Further, conductivity chromatograms of carrot extracts in absence of EDTA (with or without CaCl_2_) showed a small signal at 10 min, which is assumed to be either a matrix component or contamination from reagents or consumables (Figure S2a). However, a large signal was detected with the use of EDTA.

The added amount of EDTA in this experiment corresponded to 5 mg/mL in raw sample extracts and 2.5 mg/mL in diluted extracts (1:1 with ACN), which corresponds to the amount added when analyzing commodities with a high protein content.^5^ External calibration indicated that the signal of free EDTA in the extracts of liver and milk corresponded to ∼200 μg/mL EDTA. This was only ∼8% of the EDTA amount initially added to the samples. Still, the absolute recovery rates of Gly could not be increased above 73% in the liver. This pointed again to the hypothesis that matrix components other than free di- and trivalent cations reduced the extractability of Gly and AMPA from the liver and that these problems could not be solved with EDTA, despite its generally positive effect. Plausible candidates could be proteins that may interact with analytes via mixed-ligand metal complexes, thus reducing the amount of free metal cations.^15^

Generally, IC-MS/MS was found to be well suited for the determination of Gly and its metabolites in QuPPe extracts of various complex matrices of plant and animal origin. Specifically, EDTA did not impair the IC-MS/MS analysis of the analytes at appropriate concentrations, as used in this study. This is true for both the chromatographic separation of the analytes and their ionizations in the ESI source. Still, the amount of added EDTA should not be set higher than needed since negative effects were observed when using LC-MS/MS in individual cases.

## Supporting Information Available

Predominant microspecies of Gly, AMPA, and EDTA and their net charge (Table S1); contents of commonly occurring metal cations in the investigated matrices (Table S2); conditions of the instrument methods (Table S3); overview and details on sample preparation (Table S4); contents of commonly occurring metal cations and their corresponding amount in analytical portions in comparison with the added amount of EDTA (Table S5); overview and details on sample preparation for the model experiment with metal addition (Table S6); data adding to Figure 2 (Table S7); data on additionally investigated compounds (Table S8); recovery rates of the investigated compounds in sesame with double amount EDTA added (Figure S1); and conductivity chromatograms in carrot extract with added CaCl_2_ in the presence or absence of EDTA (Figure S2) (PDF)

## ABBREVIATIONS USED

AMPA: aminomethylphosphonic acid
AO: animal origin
dSPE: dispersive solid phase extraction
EDTA: ethylenediaminetetraacetic acid
EURL-SRM: EU Reference Laboratory for Pesticides Requiring Single Residue Methods
Gly: glyphosate
HEPA: 2-hydroxyethylphosphonic acid
IC(-MS/MS): ion chromatography (in combination with tandem mass spectrometry)
IL-IS: isotopically labeled internal standard
LC(-MS/MS): liquid chromatography (in combination with tandem mass spectrometry)
MPPA: 3-methyl-phosphinicopropionic acid
NAGly: *N*-acetyl-glyphosate
ODS: octadecylsilane (sorbent)
PO: plant origin
QuPPe (method): quick polar pesticides (method)
