# Supplementary material for: Improved Analysis of Glyphosate, Aminomethylphosphonic Acid, and Other Highly Polar Pesticides and Metabolites via the QuPPe Method by Employing Ethylenediaminetetraacetic Acid and IC-MS/MS
Source: J Agric Food Chem. 2025 Jan 15;73(4):2645–52. doi: 10.1021/acs.jafc.4c08461 (PMC11783593; doi:10.1021/acs.jafc.4c08461)
Supplement: Supplementary file 1 — jf4c08461_si_001.pdf [file jf4c08461_si_001.pdf]

Supporting Information for

Improved Analysis of Glyphosate, Aminomethylphosphonic Acid, and Other Highly Polar Pesticides and Metabolites via the QuPPe Method by Employing Ethylenediaminetetraacetic Acid and IC-MS/MS

Ann-Kathrin Schäfer[1]*, Walter Vetter[2] and Michelangelo Anastassiades[1]

[1]Chemisches und Veterinäruntersuchungsamt Stuttgart, EU-Reference Laboratory for Pesticides requiring Single Residue Methods (EURL-SRM), D-70736, Fellbach, Germany

[2]University of Hohenheim, Institute of Food Chemistry (170b), D-70599, Stuttgart, Germany

*****Corresponding author: Ann-Kathrin.Schaefer@cvuas.bwl.de

Table S1. pH-dependent microspecies of glyphosate (Gly), AMPA, *N*-acetyl-glyphosate (NAGly) and EDTA according to literature and chemicalize.com.[1,2]

**pH range**	**Predominant microspecies**	**Net charge of predominant microspecies**
	Gly, AMPA and NAGly: Values according to chemicalize.com[1]	
0 – 3.3	Gly ($-NH_2^+-$, $-PO_3H^-$, -COOH)	neutral
3.3 - 7	Gly ($-NH_2^+-$, $-PO_3H^-$, $-COO^-$)	-1
7 – 9.5	Gly ($-NH_2^+-$, $-PO_3^{2-}$, $-COO^-$)	-2
>9.5	Gly (-NH-, $-PO_3^{2-}$, $-COO^-$)	-3
0 - 7	AMPA ($-NH_3^+$, $-PO_3H^-$)	neutral
7 – 9.9	AMPA ($-NH_3^+$, $-PO_3^{2-}$)	-1
>9.9	AMPA ($-NH_2$, $-PO_3^{2-}$)	-2
1.5 – 4.2	NAGly ($-PO_3H^-$, -COOH)	-1
4.2 – 8.2	NAGly ($-PO_3H^-$, $-COO^-$)	-2
>8.2	NAGly ($-PO_3^{2-}$, $-COO^-$)	-3
	EDTA: Values according to literature[2]	
<0	H_6Y^{2+}	+2
0 – 1.5	H_5Y^+	+1
1.5 - 2	H_4Y	neutral
2 – 2.68	H_3Y^-	-1
2.68 – 6.11	H_2Y^{2-}	-2
6.11 – 10.17	HY^{3-}	-3
>10.17	Y^{4-}	-4
	EDTA: Values according to chemicalize.com[1]	
<0	H_6Y^{2+}	+2
0 – 1.6 (>50%; max. 86%)	H_5Y^+	+1
1.9 – 2.7 (>50%; max. 58%)	H_4Y	neutral
2.7 – 3.7 (>20%; max. 33%)	H_3Y^-	-1
3.3 – 4.4 (>10%; max. 19%)	H_2Y^{2-}	-2
3.8 – 5.1 (>20%; max. 35%)	HY^{3-}	-3
>4.8 (>50%)	Y^{4-}	-4
>7 (>99%)	Y^{4-}	-4

Table S2. Exemplary contents of some commonly occurring metal cations in milk, liver, lentils, sesame, wheat, cocoa beans, and cucumber according to [3] and information on a product of infant food formula (in mg/100g sample and in µg/mL in QuPPe raw extracts).

Matrix com-ponent	Milk		Liver (bovine)		Lentils		Sesame	
	Matrix [mg/100g]	QuPPe extract* [µg/mL]	Matrix [mg/100g]	QuPPe extract* [µg/mL]	Matrix [mg/100g]	QuPPe extract** [µg/mL]	Matrix [mg/100g]	QuPPe extract** [µg/mL]
**Ca**	120	600	5.8	29	65	160	780	1960
**Mg**	12	60	21	110	130	320	350	870
**Fe**	0.046	0.23	6.9	35	8.0	20	10	25
**Cu**	0.010	0.050	3.2[a)]	16	0.76	1.9		
**Zn**	0.38	1.9	4.8	24	3.4	8.5		

Matrix com-ponent	Wheat (whole grain)		Infant Formula		Cocoa (partly defatted)		Cucumber	
	Matrix [mg/100g]	QuPPe extract** [µg/mL]	Matrix [mg/100g][b)]	QuPPe extract*** [µg/mL]	Matrix [mg/100g]	QuPPe extract** [µg/mL]	Matrix [mg/100g]	QuPPe extract* [µg/mL]
**Ca**	33	83	390	390	110	290	16	80
**Mg**	3.1	7.8	36	36	410	1040	8.3	42
**Fe**	3.2	8.0	5	5	1.3	3.3	0.22	1.1
**Cu**	0.37	0.93			3.8	9.5	0.035	0.18
**Zn**	2.6	6.5	4	4	8.9	22	0.16	0.80

[a)] the copper content in liver may vary considerably depending on how much is added to the feed and on whether copper baths are used for the disinfection of hooves.[4] Copper contents in the range between 5 and 20 mg/100 g dry formula are not uncommon

[b)] information from an exemplary infant food formula product; initial declaration refers to amounts in mg per 100 mL ready to drink product using 13.7 g for the preparation

*when using 10 g sample weight

**when using 5 g sample weight

***when using 2 g sample weight

Table S3. Conditions of the IC-MS/MS instrument method.[5-7]

**IC system**	**Thermo Scientific Dionex Integrion HPIC**	
**Autosampler**	Thermo Scientific Dionex AS-AP	
**Auxilliary pump**	Thermo Scientific Dionex AXP-MS	
**MS instrument**	AB Sciex QTrap 5500	
**Column**	Thermo Scientific Dionex IonPac AS19 2x 250 mm with AG19 2x 50 mm	
**Potassium hydroxide (KOH)**	**Time**	**c (KOH) in mM**
**gradient for separation**	0	15
	8	15
	13	36
	21	36
	21.5	70
	25	70
	25.5	15
	30	15
**Flow rate (IC)**	0.3 mL/min	
**Injection volume**	5 µL of 5-fold diluted extracts in water	
**Eluent source**	Thermo Scientific Dionex EGC 500 KOH eluent generator cartridge	
**Suppressor**	Dionex ASRS 300; 2 mm	
**Temperature**	Column oven: 32 °C, Suppressor: 15 °C	
**Flow rate (Auxiliary Pump)**	0.15 mL/min ACN (MS-grade)	
**Ion source**	ESI Turbo Ion Spray, negative mode	
**Curtain gas (nitrogen)**	30 psi	
**Ion spray voltage**	-4500V	
**Gas flow**	Gas 1: 60 psi (~4.1 bar); Gas 2: 60 psi	
**Temperature of gas 2**	600 °C	

Table S4. Details on sample preparation (basic procedure and variants).

**Matrix Name**	**Cucumber**	**Carrot**	**Sesame**
**Commodity group* [8]**	1 'high water content'	1 'high water content'	4a ' high oil content and very low water content'
**Extraction approach**	'QuPPe-PO' [5]	'QuPPe-PO' [5]	'QuPPe-PO' [5]
**Sample weight**	10 g	10 g	5 g / 2 g
**Spiking absolute amount**	1 µg	1 µg	1 µg / 1 µg
**Spiking concentration**	0.1 mg/kg	0.1 mg/kg	0.2 mg/kg / 0.5 mg/kg
**IL-IS concentration**	0.2 mg/kg	0.2 mg/kg	0.4 mg/kg / 1.0 mg/kg
**Volume adjustment **** (water and/or 1 mL 10% EDTA sol.)	1 mL	1 mL	10 mL
**dSPE clean-up** (50 mg/mL raw extract) **and ACN precip. (1+1)**	no	no	yes
	**Lentils**	**Wheat**	**Cocoa Bean**
**Commodity group* [8]**	5 'high starch and/or protein content and low water and fat content'	5 'high starch and/or protein content and low water and fat content'	6 'difficult or unique commodity'
**Extraction approach**	'QuPPe-PO' [5]	'QuPPe-PO' [5]	'QuPPe-PO' [5]
**Sample weight**	5 g	5 g	5 g
**Spiking absolute amount**	1 µg	1 µg	1 µg
**Spiking concentration**	0.2 mg/kg	0.2 mg/kg	0.2 mg/kg
**IL-IS concentration**	0.4 mg/kg	0.4 mg/kg	0.4 mg/kg
**Volume adjustment **** (water and/or 1 mL 10% EDTA sol.)	10 mL	10 mL	10 mL
**dSPE clean-up** (50 mg/mL raw extract) **and ACN precip. (1+1)**	yes	yes	yes
	**Infant Food**	**Liver**	**Milk**
**Commodity group* [8]**	6 'difficult or unique commodity'	7 'meat – offal'	8 ' milk and milk products'
**Extraction approach**	'QuPPe-AO' [9]	'QuPPe-AO' [9]	'QuPPe-AO' [9]
**Sample weight**	2 g	10 g	10 g
**Spiking absolute amount**	1 µg	1 µg	1 µg
**Spiking concentration**	0.5 mg/kg	0.1 mg/kg	0.1 mg/kg
**IL-IS concentration**	1.0 mg/kg	0.2 mg/kg	0.2 mg/kg
**Volume adjustment**** (water and/or 1 mL 10% EDTA sol.)	10 mL	3 mL	1.5 mL
**dSPE clean-up** (50 mg/mL raw extract) **and ACN precip. (1+1)**	yes	yes	yes

*commodity group according to the SANTE document, which describes analytical quality control and method validation procedures for pesticide residues analysis in food and feed.[8]

**Volume of water/and or EDTA solution added to the analytical portion to obtain a total volume of ~10 mL, considering the typical water content in the matrix according to ref.[3]

Table S5. Contents of some commonly occurring metal cations from Table S1[3] and their absolute amount (n [mmol]) within the stated analytical portions of milk, liver, lentils, wheat, infant food formula, cocoa beans and sesame in the respective sample portion. For comparison, the added amount of substance of EDTA from 1 mL of a 10% solution is given.

Matrix component	Milk		Liver (bovine)		Lentils		Wheat (whole grain)	
	Matrix [mg/100g]	n in 10 g sample portion [mmol]	Matrix [mg/100g]	n in 10 g sample portion [mmol]	Matrix [mg/100g]	n in 5 g sample portion [mmol]	Matrix [mg/100g]	n in 5 g sample portion [mmol]
**Ca**	120	0.30	5.8	0.014	65	0.081	33	0.041
**Mg**	12	0.049	21	0.086	129	0.27	3.1	0.0064
**Fe**	0.046	0.000082	6.9	0.012	8.0	0.0072	3.2	0.0029
**Cu**	0.010	0.000016	3.2	0.0050	0.76	0.00060	0.37	0.00029
**Zn**	0.38	0.00058	4.8	0.0073	3.4	0.0026	2.6	0.0020
**Sum n of matrix components [mmol]**		0.35		0.13		0.36		0.053
**n addition of EDTA [mmol]**		0.34		0.34		0.34		0.34

Matrix component	Infant food formula		Cocoa (partly defatted)		Sesame (with hull)		
	Matrix [mg/100g]	n in 2 g sample portion [mmol]	Matrix [mg/100g]	n in 5 g sample portion [mmol]	Matrix [mg/100g]	n in 5 g sample portion [mmol]	n in 2 g sample portion [mmol]
**Ca**	387	0.19	114	0.14	783	0.98	0.39
**Mg**	36	0.030	414	0.85	347	0.71	0.29
**Fe**	5	0.0018	1.3	0.0012	10	0.0089	0.0036
**Cu**			3.8	0.0030			
**Zn**	4	0.0012	8.9	0.0068			
**Sum n of matrix components [mmol]**		0.23		1.0		1.7	0.68
**n addition of EDTA [mmol]**		0.34		0.34		0.34	0.34

Table S6. Details on metal addition for the model experiment.

	Addition of Ca^{2+} absolute [mg]/[mmol]	Addition of $CaCl_2$ [mg]	Examples
	**Carrot + Ca^{2+}**		
**#1**	0/0	0	
**#2**	5/0.125	14	
**#3**	10/0.250	28	10 g milk: ~12 mg Ca
**#4**	15/0.375	42	
**#5**	20/0.500	56	
**#6**	25/0.625	69	
	**Carrot + Fe^{3+}**		
	Addition of Fe^{3+} absolute [µg]/[mmol]	Addition of $FeCl_3$ of 10 mg/mL sol. [µL]	
**#1**	0/0	0	
**#2**	500/0.00895	50	10 g bovine liver: 700 µg Fe
**#3**	1000/0.0179	100	

Table S7: Detailed recovery data in addition to **Fig. 2**.

	**Without IL-IS correction**				**After IL-IS correction**			
	without EDTA (corresponding to blue bars in **Fig. 2**)		with EDTA (corresponding to green bars in **Fig. 2**)		without EDTA (corresponding to blue diamonds in **Fig. 2**)		with EDTA (corresponding to green diamonds in **Fig. 2**)	
	**Average recovery**	RSD	**Average recovery**	RSD	**Average recovery**	RSD	**Average recovery**	RSD
				**Glyphosate**				
**Cucumber**	94	1.7	92	4.5	102	0.5	101	0.2
**Milk**	29	9.3	93	1.6	101	2.2	96	1.5
**Liver**	39	2.0	73	2.3	106	0.8	101	2.3
**Sesame 2g**	9	2.8	87	3.8	101	6.8	101	2.0
**Sesame 5g**	6	4.9	54	2.2	105	8.4	104	2.3
**Lentils**	42	2.7	57	3.8	104	0.4	100	3.7
**Cocoa**	5	11	50	2.3	95	9.4	101	2.3
**Wheat**	16	10	60	6.4	102	1.3	97	1.0
**Infant food**	3	9.2	71	3.7	99	1.3	101	0.1
				**AMPA**				
**Cucumber**	91	3.5	94	2.9	100	0.8	99	0.7
**Milk**	18	7.4	93	0.5	89	4.4	98	0.3
**Liver**	22	3.1	90	3.7	98	2.2	102	1.0
**Sesame 2g**	7	2.3	86	5.1	87	1.4	98	0.9
**Sesame 5g**	4	3.0	57	2.5	81	5.4	99	1.2
**Lentils**	27	5.2	60	1.6	95	1.7	96	1.6
**Cocoa**	-	-	47	1.3	-	-	101	2.6
**Wheat**	53	3.2	74	0.8	101	0.9	99	1.2
**Infant food**	10	9.6	73	3.7	99	4.7	98	1.6
				**NAGly**				
**Cucumber**	101	0.5	98	0.5	100	0.5	100	1.1
**Milk**	95	2.7	91	1.9	94	4.0	99	1.7
**Liver**	83	0.7	66	1.4	101	2.0	100	1.2
**Sesame 2g**	82	3.5	101	3.6	100	3.1	99	1.7
**Sesame 5g**	61	1.0	83	1.4	92	1.4	100	1.9
**Lentils**	88	2.5	73	4.4	98	0.7	95	4.6
**Cocoa**	80	2.7	89	2.3	95	5.7	97	0.3
**Wheat**	28	1.0	84	1.6	98	1.2	99	0.2
**Infant food**	71	1.0	84	2.4	99	1.2	100	0.4

Table S8: Detailed recovery data of other mentioned polar analytes.

	**Without IL-IS correction**				**After IL-IS correction**			
	without EDTA		with EDTA		without EDTA		with EDTA	
	**Average recovery**	RSD	**Average recovery**	RSD	**Average recovery**	RSD	**Average recovery**	RSD
	**Glufosinate**							
**Cucumber**	100	2.1	96	1.1	99	0.2	101	0.3
**Milk**	99	3.2	94	2.0	102	1.6	102	1.0
**Liver**	92	2.4	88	4.3	100	0.7	99	1.6
**Sesame 2g**	110	2.4	102	4.8	104	2.9	101	2.8
**Sesame 5g**	98	2.3	93	3.7	99	1.8	101	2.4
**Lentils**	94	4.8	77	4.2	101	0.2	98	4.4
**Cocoa**	97	1.5	89	0.6	102	2.1	100	1.5
**Wheat**	82	2.8	83	0.2	99	0.5	98	0.3
**Infant food**	89	1.5	80	2.8	100	0.5	101	0.2
	**3-methyl-phosphinicopropionic acid (MPPA)**							
**Cucumber**	101	3.0	97	1.2	100	0.6	100	0.7
**Milk**	105	3.7	96	0.2	102	1.9	102	1.2
**Liver**	98	2.9	85	3.3	99	1.6	98	0.8
**Sesame 2g**	106	3.8	101	1.5	99	0.9	98	1.7
**Sesame 5g**	97	3.6	94	0.8	100	1.0	98	2.3
**Lentils**	97	3.7	88	1.1	99	0.4	97	0.8
**Cocoa**	105	3.5	97	1.2	103	1.2	98	1.1
**Wheat**	80	1.9	90	1.3	101	0.9	99	0.9
**Infant food**	93	1.2	87	2.9	100	0.3	99	1.1
	***N*-acetyl-glufosinate**							
**Cucumber**	102	1.1	99	0.5	77	11	100	0.6
**Milk**	95	5.0	97	1.2	96	0.3	103	0.7
**Liver**	98	0.9	84	1.4	104	4.5	101	0.7
**Sesame 2g**	108	3.8	104	3.1	101	3.2	103	4.6
**Sesame 5g**	100	4.2	97	3.0	102	4.5	103	2.0
**Lentils**	99	2.9	87	2.9	97	4.5	94	1.4
**Cocoa**	107	2.0	97	1.7	105	1.6	102	2.6
**Wheat**	83	0.2	96	0.7	99	0.3	99	0.3
**Infant food**	91	0.3	88	1.8	99	1.2	101	0.6
	**Fosetyl**							
**Cucumber**	103	2.2	97	1.4	101	1.1	99	1.0
**Milk**	103	3.9	96	2.1	102	0.5	98	1.5
**Liver**	102	0.8	92	2.0	99	1.3	102	1.2
**Sesame 2g**	99	5.0	98	2.0	98	2.0	96	2.0
**Sesame 5g**	94	0.5	92	3.6	100	0.5	100	0.8
**Lentils**	95	1.1	94	2.4	100	1.1	100	0.7
**Cocoa**	102	1.5	97	0.6	101	1.3	99	0.7
**Wheat**	85	6.2	94	1.1	99	0.5	102	1.0
**Infant food**	98	3.2	91	1.8	101	0.3	100	1.1

	Without IL-IS correction				After IL-IS correction			
	without EDTA		with EDTA		without EDTA		with EDTA	
	**Average recovery**	RSD	**Average recovery**	RSD	**Average recovery**	RSD	**Average recovery**	RSD
**Ethephon**								
**Cucumber**	104	4.0	96	0.9	102	2.2	98	1.5
**Milk**	94	2.3	88	1.6	96	2.4	100	3.1
**Liver**	80	1.2	71	2.8	100	1.8	100	1.4
**Sesame 2g**	99	3.7	93	1.1	98	2.2	100	1.7
**Sesame 5g**	86	3.4	84	1.8	101	1.4	102	3.2
**Lentils**	83	0.3	75	2.3	101	1.6	99	2.1
**Cocoa**	96	1.3	94	1.4	99	1.5	100	0.9
**Wheat**	70	3.4	80	0.1	102	0.4	102	0.8
**Infant food**	90	0.8	86	1.8	100	1.4	100	0.5
**2-hydroxyethylphosphonic acid (HEPA)**								
**Cucumber**	102	3.7	98	2.3	99	0.7	101	1.1
**Milk**	96	3.9	96	0.7	102	2.4	99	1.0
**Liver***	-	-	-	-	-	-	-	-
**Sesame 2g**	104	4.5	100	7.8	100	1.7	98	4.6
**Sesame 5g**	93	2.6	88	6.3	103	1.6	115	4.2
**Lentils**	89	5.4	87	2.8	98	0.3	104	2.7
**Cocoa**	101	0.7	102	4.9	97	4.1	106	3.1
**Wheat**	77	2.2	88	0.5	100	0.1	99	0.6
**Infant food**	92	1.2	86	2.6	100	0.1	101	0.1

*evaluation was not possible due to high background levels

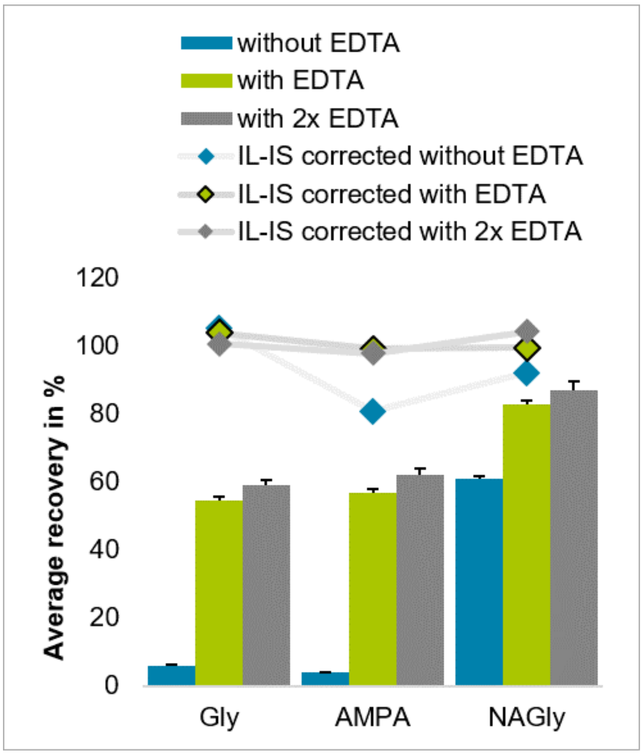

Fig. S1. Average recovery rates (results of ‘experiment 2’) in sesame with 5 g sample weight achieved for glyphosate, AMPA and NAGly without EDTA with 0.34 mmol EDTA and with 0.68 mmol EDTA (2x EDTA) as well as with or without IL-IS. Average absolute recovery rates are shown in bars and the average IL-IS-corrected recovery rates are shown as diamonds

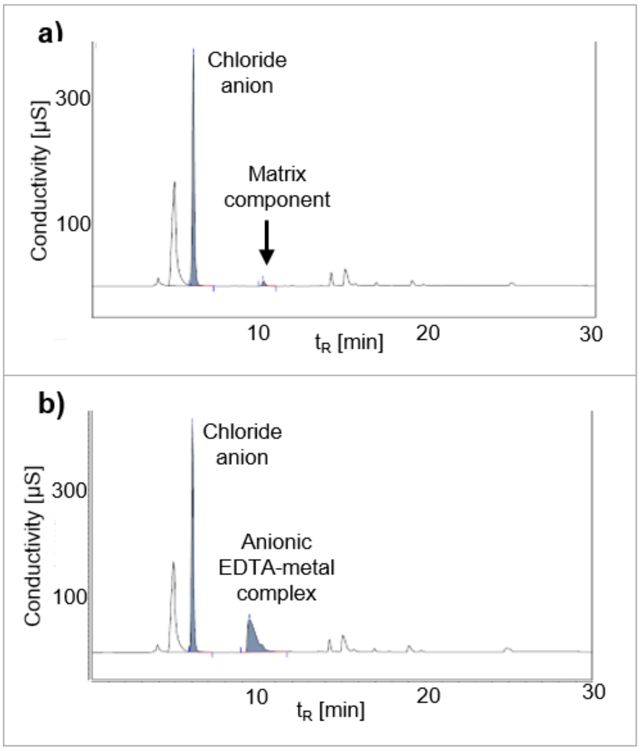

Fig. S2. Conductivity chromatogram of a calibration standard in carrot extract following 5 fold dilution in water with (**a**) addition of 10 mg Ca^{2+} as $CaCl_2$ and (**b**) addition of 10 mg Ca^{2+} as $CaCl_2$ and EDTA during extraction

References

[1] Chamicalize.com. URL (https://chemicalize.com/welcome) (Accessed Aug 2024).

[2] Harvey, D. Titrimetric Methods of Analysis. In *Model Analytical Chemistry*; McGraw-Hill Higher Education. Publisher: Smith, J.M., United States, 2000, pp. 314-331.

[3] Souci; Fachmann; Kraut. Food Composition and Nutrition Table, Online Database. URL (https://www.sfk.online/#/home) (Accessed May 2024).

[4] Strickland, J.M:; Herdt, T.H.; Sledge, D.G.; Buchweitz, J.P. Short communication: Survey of hepatic copper concentrations in Midwest dairy cows. JDS. **2019**; *102*, 4209-4214.

[5] Anastassiades, M.; Schäfer, A.-K.; Kolberg, D.I.; Eichhorn, E.; Dias, H.; Benkenstein, A.; Zechmann, S.; Mack, D.; Wildgrube, C.; Barth, A.; Sigalov, I.; Goerlich, S.; Dörk, D.; Cerchia, G. Quick Method for the Analysis of Highly Polar Pesticides in Food Involving Extraction with Acidified Methanol and LC- or IC-MS/MS Measurement I. Food of Plant Origin (QuPPe-PO-Method), Version 12.2, 21.12.2023. URL (https://www.eurl-pesticides.eu/userfiles/file/EurlSRM/EurlSrm_meth_QuPPe_PO_V12_2.pdf) (Accessed Aug 2024).

[6] Wachtler, A.-K.; Wildgrube, C.; Mack, D.; Barth, A.; Anastassiades, M.; Scherbaum, E.; Vetter, W. Analysis of Highly Polar Pesticides in Food of Plant and Animal Origin by IC-MS/MS: 13th EPRW 2020, PD-87. URL (https://www.eurl-pesticides.eu/userfiles/file/EurlSRM/EPRW%202020%20-%20PD87.pdf) (Accessed May 2024).

[7] Schäfer, A.-K.; Vetter, W.; Anastassiades, M. Analysis of Highly Polar Pesticides in Food of Plant and Animal origin by Ion Chromatography and Tandem-Mass Spectrometry with Emphasis on addressing adverse Effects caused by Matrix Co-Extractives. Anal. Bioanal. Chem. **2024**, *416*, 4503-4517.

[8] SANTE 11312/2021 v2: Analytical quality control and method validation procedures for pesticides residue analysis in food and feed. Implemented by 01/01/2024.

[9] Anastassiades, M.; Wachtler, A.-K.; Kolberg, D.I.; Eichhorn, E.; Benkenstein, A.; Zechmann, S.; Mack, D.; Barth, A.; Wildgrube, C.; Sigalov, I.; Goerlich, S.; Dörk, D.; Cerchia, G. Quick Method for the Analysis of Numerous Highly Polar Pesticides in Food Involving Extraction with Acidified Methanol and LC-MS/MS Measurement II. Food of Animal Origin (QuPPe-AO-Method), Version

3.2, 14.05.2019. URL (https://www.eurl-pesticides.eu/userfiles/file/meth_QuPPe_AO_V3_2.pdf) (Accessed May 2024).
